# Evidence of Gas Phase Glucosyl Transfer and Glycation in the CID/HCD-Spectra of S-Glucosylated Peptides

**DOI:** 10.3390/ijms25137483

**Published:** 2024-07-08

**Authors:** Alicja K. Buchowiecka

**Affiliations:** Institute of Molecular and Industrial Biotechnology, Faculty of Biotechnology and Food Sciences, Lodz University of Technology, Stefanowskiego 2/22, 90-537 Łódź, Poland; alicja.buchowiecka@p.lodz.pl

**Keywords:** glycoproteomics, S-glycosylation, gas phase glycosyl transfer, glycation, Amadori rearrangement

## Abstract

Protein cysteine S-glycosylation is a relatively rare and less well characterized post-translational modification (PTM). Creating reliable model proteins that carry this modification is challenging. The lack of available models or natural S-glycosylated proteins significantly hampers the development of mass-spectrometry-based (MS-based) methodologies for detecting protein cysteine S-glycosylation in real-world proteomic studies. There is also limited MS-sequencing data describing it as easier to create synthetic S-glycopeptides. Here, we present the results of an in-depth manual analysis of automatically annotated CID/HCD spectra for model S-glucopeptides. The CID spectra show a long series of y/b-fragment ions with retained S-glucosylation, regardless of the dominant *m*/*z* signals corresponding to neutral loss of 1,2-anhydroglucose from the precursor ions. In addition, the spectra show signals manifesting glucosyl transfer from the cysteine position onto lysine, arginine (Lys, Arg) side chains, and a peptide N-terminus. Other spectral evidence indicates that the N-glucosylated initial products of transfer are converted into N-fructosylated (i.e., glycated) structures due to Amadori rearrangement. We discuss the peculiar transfer of the glucose oxocarbenium ion (Glc+) to positively charged guanidinium residue (ArgH+) and propose a mechanism for the gas-phase Amadori rearrangement involving a 1,2-hydride ion shift.

## 1. Introduction

Cellular glycosylation of proteins is an enzyme-directed process that leads to diverse protein-glycan structures, which are responsible for driving particular biological functions [1]. Most glycans are assembled from a limited set of reducing monosaccharides linked by O, O-acetal bridges, which are themselves attached through O- or N-glycosidic bonds to side chains of serine/threonine (Ser/Thr) or asparagine (Asn) residues at specific locations on the polypeptide chains [2]. These major types of glycosidic bonds have distinctive properties, which can be analyzed using well-established MS-based technologies [3].

The chemical synthesis of glycans and glycoconjugates has enabled the development of modern glycoproteomics by providing reference samples for systematic biological studies [4]. In general, the synthesis of complex glycan intermediates relies on the transfer of the glycosyl residue from a selected, activated donor to a saccharide serving as an acceptor molecule. In classic chemical strategies, the acceptor has a target hydroxyl group accessible for the formation of a glycosidic linkage, whereas the reducing end of the growing glycan chain is orthogonally protected to permit the eventual assembly of final protein glycoconjugates [5]. Another original approach achieves control over the regio- and stereochemistry of glycan synthesis by intramolecular glycosyl transfer. However, this strategy is not frequently used due to the great difficulty of acquiring sophisticated molecules concurrently exhibiting glycosyl donor and glycosyl acceptor functionalities [6].

Thioglycosides are favorable glycosyl donors due to their efficient activation by various thiophilic promoter systems [7]. Chemical glycosylation is accomplished in extra-dry organic solvents to protect the activated donors from hydrolysis. In addition to the desired glycosylation product, the reaction typically generates a byproduct with no nucleophilic activity—the thioaglycon. The thioaglycon often remains tightly bound to the promoter used for activation and should be removed from the reaction mixture.

In contrast to extensively studied, structurally complex N- and O-glycosylated proteins, S-glycosylation involving protein cysteines is considered a rare post-translational modification (PTM). The S-linked glycans identified to date are mono-saccharides or short oligo-saccharides made of Glc, Gal, and GlcNAc residues. The distinctive mono-ADP-S-ribosyl unit attached to proteins can also be categorized as S-glycans [8].

The experimental results of MS/MS-sequencing of glycopeptides show that S-glycosidic bonds in CID-type MS fragmentation are more stable than O-glycosidic linkages. This feature can help to determine the location of S-glycosylated cysteines in many proteins [9,10,11,12]. For example, by employing a routine proteomic workflow with final LC-ESI tandem mass spectrometry, researchers have detected S-glycosylated catalytic cysteines transiently occurring in glycosyl-enzyme intermediates. Based on these findings, a retaining mechanism for catalysis of two mutant glycosyltransferases has been proposed [13]. Nonetheless, detecting low-level S-linked glycosylation among ubiquitous O- and N-glycosylated proteins remains challenging and requires new tailored analytical methods [14].

Our previous work described a chemically modified lysozyme (P-00698) randomly S-glucosylated at cysteine positions available in the protein molecule [14]. The locations of the S-modified sites were detected directly using routine mass spectrometric methods and indirectly after specific S-tagging of the S-glucosylated positions. As a result, a large library of CID/HCD fragmentation spectra was created for the model S-glucosylated and S-tagged peptides. Here, we present the results of a manual analysis of complex MS/MS spectra of selected S-glucosylated peptides from the previously created library. In the CID spectra, signals showing 162 Da neutral loss from the precursor ions dominate, accompanied by a series of y/b-fragment ions retaining intact S-glucosyl moieties. In addition, numerous deciphered signals provide spectral evidence of glucosyl transfer from cysteine onto Lys/Arg side chains and the N-terminals of the fragmenting peptides. Progressing the S→N glucosyl transfer leads to the formation of N-glucosylated derivatives and N-fructosylated derivatives known as Amadori products [15]. We propose hypothetical mechanisms for these gas-phase transformations.

## 2. Results

The present study builds on our previous work [14], in which CID/HCD fragmentation spectra of S-glucopeptides were identified using the Byonic sequence search engine. The created CID/HCD spectral library contained 90 unique sequences of S-glucopeptides. In the present study, a set of spectra from the CID/HCD spectral library was selected for meticulous manual analysis. The selected spectra included precursor ions of charge state z = +2 or z = +3, with one S-glucosylated cysteine position. The omitted spectra possessed long sequences bearing more modifications and a precursor ion charge state z > 3.

Initial manual analysis was focused on identifying *m*/*z* signals corresponding to glucosyl oxocarbenium ion (Glc+, 163 Da) transfer from the Cys residue onto side chains of other amino acid residues.

### 2.1. General Characteristics of S-Glucopeptides MS-Fragmentation

The CID spectra showed a characteristic strong signal representing 1,2-anhydroglucose neutral loss (162 NL) from the +2 charged precursors (Figures S1–S9). Occasionally, glucosyl loss (Glc+, 163 Da) occurred from the +3-charged precursor ion, leading to a reduction of its charge state, as illustrated in Figure S3. Other signals of neutral losses from precursor ions, denoted as [M + zH^+^]/z, show the loss of hexose (180 NL), pentose (150 NL), tetrose (120 NL), and triose (90 NL). The CID spectra of S-glucosylated peptides signals show the loss of one (18 NL), two (36 NL), or three (54 NL) water molecules.

The automatically annotated long series of b/y fragment ions with retained C[S-Glc]-modification were typical for most of the CID spectra and proved the moderate stability of the modification. Manual searching revealed the presence of b/y fragment ions with no Cys in their sequences but bearing a mass gain of 162 Da. This was linked to Glc+ ion transfer from cysteine to other amino acid residues within fragmenting peptides. In particular, [b+162]+/++ fragment ions corresponding to short N-terminal sequences and [y+162]+/++ comprising Lys or Arg on C-termini were detected. Unexpectedly, fragment ions denoted as [y/b+162+162−losses]+/++ were also revealed. This implies the transfer of two Glc+ ions onto fragments with no Cys residue in their sequences.

In the HCD spectra, [M + zH^+^ − 162]/z ions were weak or absent. A long series of y-type fragment ions from +2 and +3 charged precursors was observed, as presented in Section 2.3.2.

### 2.2. Literature-Based Characteristics of Peptidyl N-Glucosylated Arg and Lys MS-Fragmentation

Although not previously described in the literature, Glc+ ion migration onto Lys primary amine groups or peptide N-termini in a gas phase seemed plausible and worth further exploration. Yet, a similar glucosyl transfer onto protonated arginine residues raised questions about the mechanism of transformation. The assumed formation of N-glucosylated arginine with subsequent Amadori rearrangement would lead to the cognate isobaric product (i.e., 1-amino-1-deoxy-D-fructoside), which should be distinguishable in the CID spectra [16]. Therefore, we conducted a thorough literature survey on the use of mass spectrometry in protein glycation studies. In particular, we focused on published data referring to the diagnostic fragmentation features of peptidyl N-glucosides, N-fructosides, and selected N-glycopeptides. The results are summarized in Table 1.

**Figure 1:**
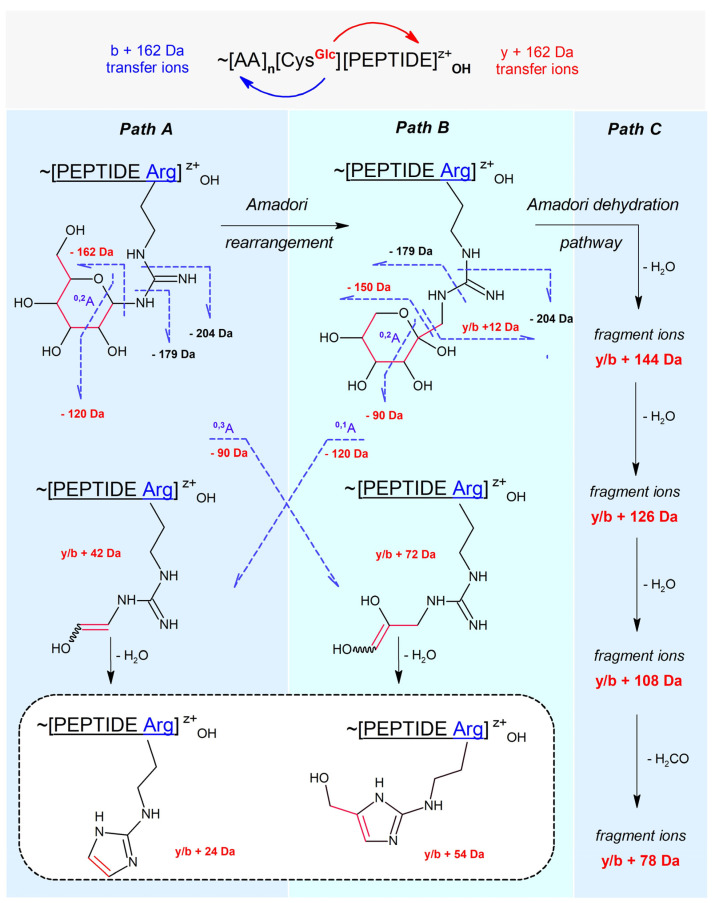
CID fragmentation pathways of N-glucosylated peptides containing Arg or Lys.

Our review of the literature data enabled us to establish a general map of N-glucosylated peptidyl arginine CID/HCD fragmentation, which is presented in Figure 1. This map is applicable also to the CID fragmentation of N-glucosylated peptidyl lysine—i.e., if the pictured guanidinium group is replaced by the primary amino group. Then, 204 Da, 54 Da, and 24 Da diagnostic ions are not expected. The distinguished paths A, B, and C illustrate the main fragmentation routes, termed as follows:

- Path A: peptidyl N-glucoside fragmentation (Schiff base fragmentation path)
- Path B: peptidyl N-fructoside fragmentation (Amadori product fragmentation path)
- Path C: peptidyl N-fructoside dehydration (Amadori product dehydration path)

N-glucosides of primary amines are cyclic hemi-aminals (in the open form known as Schiff bases). Under physiological conditions, they hydrolyze easily or undergo Amadori rearrangement catalyzed by protic acids into isobaric N-fructosides (i.e., 1-amino-1-deoxy -D-fructosides) [16,17]. To the best of our knowledge, the gas phase Amadori rearrangement of N-glucosylated peptides (under CID/HCD fragmentation conditions) has not been described previously. However, it is worth highlighting earlier studies that describe the CID fragmentation patterns of N-glucosylated asparagine in peptides [18] and discuss ^0,2^A n cross-ring cleavage as a general diagnostic tool for glycan assignment in glycoconjugate mixtures [19].

The detected signs of glucosyl (Glc+) transfer onto protonated arginine residue (ArgH+) led us to review the literature on the application of mass spectrometry in studies of protein arginine glycosylation [20]. It is known that arginine residue in vivo can be N-glucosylated, N-GlcNAcetylated, N-rhamnosylated [21], and mono-ADP-N-ribosylated due to enzyme-catalyzed processes [22]. These modifications are prone to glycan and carbodiimide (42 Da, CN_2_H_2_) loss during CID. Therefore, it seemed that the 204 NL signal (162 Da + carbodiimide = 204 Da) might be a useful diagnostic marker for detecting N-glucosylated arginine in the CID spectra of our S-glucosylated peptides.

Under physiological conditions, the primary amino groups within proteins may follow non-enzymatic condensation with aldehydes, typically reducing sugars such as glucose. This reaction proceeds through an acyclic imine (Schiff base), followed by Amadori rearrangement to form so-called glycation products (e.g., N-fructosylated derivatives) [15,23]. Protein glycation can alter biomolecule natural functionalities and ultimately lead to abnormal biological processes. This important topic has been discussed in a large number of studies. In many of those studies, mass spectrometry was instrumental in detecting glycation structures [24,25,26,27,28].

### 2.3. Detailed Analysis of MS-Fragmentation Spectra for S-Glucopeptides

Fragmentation spectra of the following tryptic S-glucosylated peptides were subjected to detailed manual analysis:

- C[+162.]KGTDVQAWIR
- C[+162]ELAAAMK    (Tables S1 and S2; Figures S5 and S6)
- RC[+162]ELAAAMK    (Table S3; Figures S7 and S8)
- WWC[+162]NDGR    (Table S4; Figures S9 and S10)
- SLGNWVC[+162]AAK   (Table S5)

These sequences have C-terminal lysine or arginine and single S-glucosylated cysteine located at the N-termini or within the peptide chain. As the exemplary sequence, C[+162.]KGTDVQAWIR was selected due to the accessibility of CID and HCD spectra for both +2 and +3 charged precursors. The results of the manual analysis are presented in Section 2.3, corresponding to the logic of Figure 1. The centrally positioned peptide sequence guides the reader over the data arranged in vertical and horizontal lines. The b- and y-ions were automatically annotated. Their total abundance was calculated separately for each ion type and considered as 100%, or the value of 1. Manually deciphered fragment ions arranged in columns represent entities of the same cleavage position but differing by mass loss or gain. Appropriately marked, diagnostic fragment ions derived from the decomposition of b-ions are placed in the upper rows. They indicate gas phase events on the precursor N-terminus. Changes in the precursor C-terminus are manifested by y-type diagnostic ions, analogically ordered in the lower rows. Their total abundance was expressed as the relative abundance versus the total abundance calculated for y ions.

#### 2.3.1. CID-Fragmentation Analysis of C[+162.]KGTDVQAWIR Sequence

**Figure 2:**
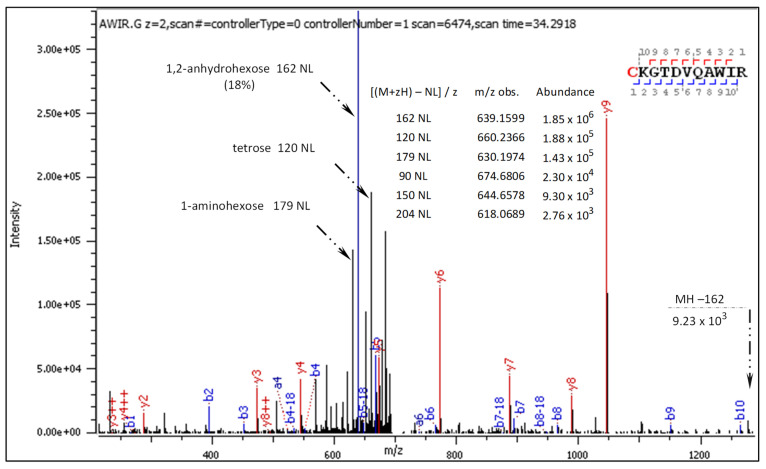
CID MS/MS spectrum of doubly charged C[+162.]KGTDVQAWIR.

Fragment ions revealed in the CID spectrum of the +2 charged precursor (Figure 2) are presented in Table 2 and Table 3. The long series of signals corresponding to this particular type of diagnostic ions provides evidence of molecular transformation processes ongoing in the gas phase, which are explained in an additional table (Table 4).

As can be seen in Table 3, the fragment ions b2 + 162, b3 + 162, and b8 + 162 carry an additional hexosyl residue, most likely attached either to the lysine residue or to the cysteine N-terminus, apparently due to an intermolecular glucosyl transfer. The diagnostic ions b1 + 144, b1 + 126, and b1 + 108 likely indicate an S→N glucosyl shift followed by Amadori rearrangement to N-fructosylated cysteine.

The general characteristics of the CID fragmentation patterns for the S-glucosylated peptides were presented in the Section 2.1. Figure 2 displays multiple neutral loss signals from the +2 charged precursor with dominating 162 NL. The relatively strong 179 NL peak (1.45 × 10^5^) corresponds to 1-aminoglucose loss from N-glucosylated arginine. The less abundant 204 NL (2.76 × 10^3^) indicates the combined loss of 1,2-anhydroglucose and carbodiimide.

Diagnostic fragment ions from the CID spectrum of the +3 charged precursor are collected in Table 5 and Table 6 and commented on in a table (Table 7).

**Figure 3:**
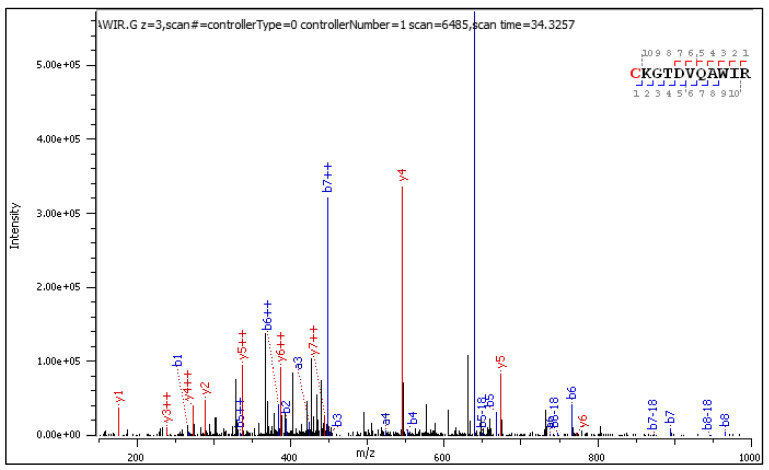
CID MS/MS spectrum of triply charged C[+162.]KGTDVQAWIR.

CID fragmentation of the +2 and +3 charged C[+162.]KGTDVQAWIR delivers similar sets of diagnostic fragment ions, experiencing a mass loss or gain according to paths A, B, and C from Figure 1. Yet, in the CID spectrum of the +3 precursor shown in Figure 3, singly charged diagnostic ions are accompanied by longer series of the same but doubly charged diagnostic ions with higher abundance.

#### 2.3.2. HCD-Fragmentation Analysis of the C[+162.]KGTDVQAWIR Sequence

**Figure 4:**
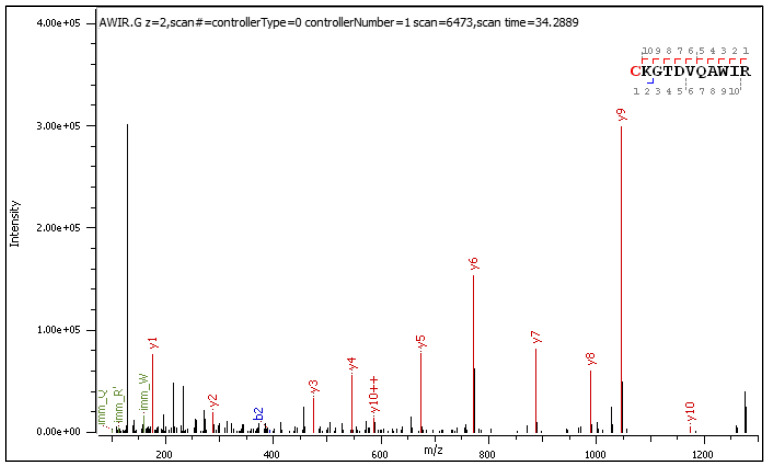
HCD MS/MS spectrum of doubly charged C[+162.]KGTDVQAWIR.

**Figure 5:**
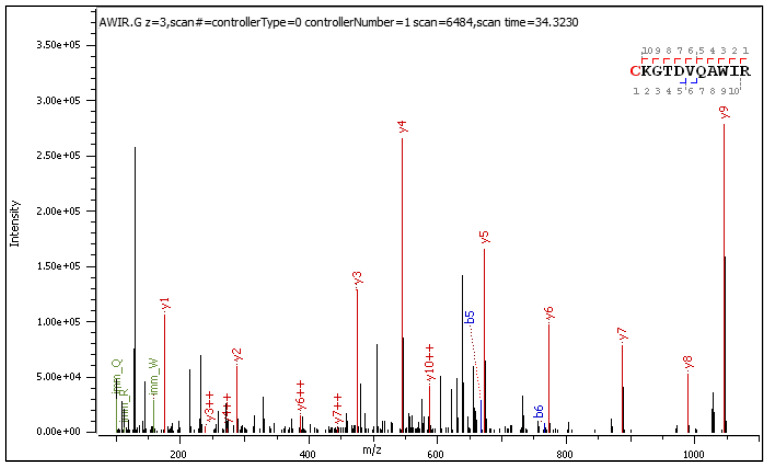
HCD MS/MS spectrum of triply charged C[+162.]KGTDVQAWIR.

A few ions confirming glucosyl transfer and subsequent Amadori rearrangement populate the HCD spectra of the +2 and +3 charged precursors (Figure 4 and Figure 5, respectively). The fragment ions containing a hydroxymethyl-imidazole ring [y+54]+/++ are slightly more abundant than the others. Manual analysis of selected peptides with N-terminal lysine and S-glucosylated cysteine located within the sequence confirmed all observations and conclusions presented in the previous sections. These results are presented in the Supplementary Materials (Tables S6–S8, Figure S11 and Figure S12).

## 3. Discussion

The CID spectra of S-glucosylated peptides reveal the presence of automatically assigned principal fragment ions along with numerous unidentified *m*/*z* signals, including high-intensity peaks. Manual examination of the spectra enabled us to assign the fragment ions to many previously undefined *m*/*z* signals. Their presence sheds light on the side transformations concurrent with the MS/MS-sequencing process.

We identified fragments [y/b+162]+/++, recognized as linked to the putative migration of glucosyl oxcarbenium ion (Glc+, i.e., 163 Da) from the Cys to Lys and Arg residues. We further identified the signal of a general notation [y/b+162−losses]+/++, corresponding to initially formed N-glucosides, which can further undergo Amadori rearrangement into isobaric N-fructosides (Figure 1). We detected doubly glycosylated fragment ions, labeled as [y/b+162+162−losses]+/++, indicating ongoing Glc+ transfers between ion fragments.

The mechanisms of these molecular transformations in a gas phase demand explication in the context of analogous phenomena reported in the literature for ion/ion and ion/molecule reactions [29,30]. The postulated transfer of the glucose oxocarbenium ion Glc+ onto the guanidinium moiety of arginine (ArgH+) seems puzzling, for it would require difficult deprotonation of (ArgH+), enabling N-glucosylation of the uncharged arginine side chain.

### 3.1. Reported Spectral Evidence of Gas Phase Glycosyl Transfer

The following examples are drawn from mass spectrometric studies on unique gly-cans derived from various glycoconjugates. Currently, glycan rearrangement is observed via the migration of small monosaccharides to other intra-glycan positions, using CID analysis. In such cases, the glycan molecule plays a double function as the glycosyl donor and acceptor. Intra-molecular xylose migration has been observed using tandem mass spectrometry of N-linked glycans [31]. In studies on the Lewis X (Lex) and blood group antigen H-2, the CID-induced migration of fucose assisted by mobile proton led to the formation of a new O-glycosidic linkage [32]. Another intriguing outcome relates to CID-MS/MS analysis of a synthetic neo-glycolipid. As a result of an intramolecular mechanism of O-to-C glycosyl transfer, a C-glycosylated cholesterol derivative formed [33].

### 3.2. Glucosyl Transfer Evidence in CID/HCD Spectra of S-Glucopeptides

Tryptic peptides must cover a long analytical pathway, from protein digestion through chromatographic separation and complex ESI-MS/MS spectra acquisition, to become sequenced [34,35]. In the dry gas phase, charged peptides are flexible and continuously shape their conformations via intramolecular hydrogen bonds, salt bridges, hydrophobic interactions, and collisional interactions [36,37]. In aqueous conditions, the flexibility of peptides can be determined by measuring their end-to-end collision frequency [38]. Peptides’ conformational motions in a gas phase can be computed using the eBGF algorithm [39].

In the present study, we selected CID/HCD spectra of S-glucosylated peptides containing the C[S-Glc] modification as a glycosyl ion [Glc+] source and diverse reactive groups located in their side chains. We assumed that under CID conditions the S-glycosidic bonds undergo “trashless” activation due to multiple collisions of the peptide with neutral gas molecules, as well as self-collisional interactions. As a result, a reactive transient ion pair, [Glc+/anh-Glc+][: S-Cys][Peptide]^z+^_OH_, composed of Glc-oxocarbenium ion/protonated 1,2-anhydro-glucose [40] and peptidyl thiolate, may appear. This contact ion pair would be held together by electrostatic attraction. In most cases, collisional activation leads to a neutral loss of 1,2-anhydro-glucose (162 Da) from the precursor ion. The 163 *m*/*z* signal of Glc+ may also appear in the MS/MS spectra.

The existing transient ion pair can collide with its conformationally accessible side chains, bearing nucleophilic/electrophilic functional groups—e.g., those located at the peptide C- or N-terminus. In anhydrous gaseous environments, the cysteine thiolate ion, [: S-Cys][Peptide]^z+^_OH_, exhibits variable nucleophilicity and basicity, which depend on the peptide sequence and conformation. Therefore, we postulate that in favored spatial arrangements, self-collisional peptide interactions may result in glucosyl transfer from Cys to side chains of Lys and Arg, or N-terminal amine groups. Such a collisional state brings together the critical structural elements of the glycopeptide—i.e., the glucosyl donor group, acceptor side chains, and groups involved in proton transfer (-NH_2_, -SH, -COOH). Thus, in certain respects, such a collisional state resembles the catalytic center environment of some glycosyltransferases.

**Figure 6:**
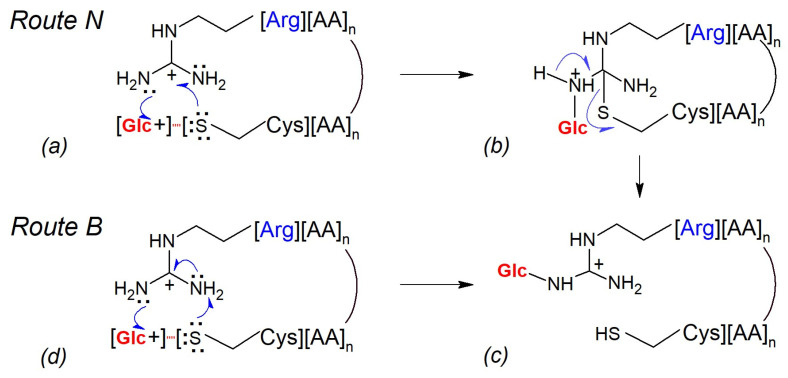
Hypothetical mechanisms of gas-phase glucosyl transfer from Cys residue to the guanidinium group of Arg residue. (**a**) Peptidyl-cysteine thiolate acts as a nucleophile; thioeter cyclopeptide formation, Glc+ capture; (**b**) N-glucosylated thioether cyclopeptide ring opening; (**c**) N-glucosylated charged peptidyl arginine (Arg+) formation; (**d**) Peptidyl-cysteine thiolate acts as a base; guanidinium group deprotonation, Glc+ capture.

Generally, the stereochemistry of collisions and the energies of the colliding partners may control the mechanisms of glucosyl transfer to the Arg guanidinium end. Two routes, denoted Route N and Route B, are postulated. In Route N, the peptidyl thiolate acts as a catalytic nucleophile (N), whereas in following Route B, it behaves as a catalytic base (B). Figure 6 illustrates these two possible mechanisms of arginine N-glycosylation of peptides in a gas phase, with route B also applying for N-glycosylation of the protonated lysine and the peptide N-terminus.

1. Route N: Cysteine thiolate acts as a catalytic nucleophile

We postulate that the [Glc+/anh-Glc+][: S-Cys][Peptide]^z+^_OH_ ion pair can collide with a guanidinium group of the C-terminal arginine (ArgH+). Steric interactions and Columb attraction/repulsion forces affect the collisional transient structure and impact the S→N glucosyl transfer process.

The guanidinium function of arginine is a planar resonance-stabilized structure comprising a central carbon atom of sp2 hybridization, which is linked to three nitrogen atoms by single C-N bonds [41]. The approaching thiolate anion neutralizes the positive charge of guanidinium carbon and changes the orbital hybridization of both carbon and nitrogen atoms. The resulting uncharged transient thioether cyclopeptide has tetrahedral carbon connected to three sp3-hybridized amine groups. This sterically crowded and electron-rich structural element traps the proximate Glc+ ion to form a C1-N+ glycosidic linkage with an accessible primary amine group. The positive charge of the created transitional structure is located on a single nitrogen atom and is not resonance-stabilized. Finally, the reaction of a thioether ring opening liberates the side chain’s cysteine thiol and restores the energetically favorable planar guanidinium N-glucosylated function.

In summary, the proposed mechanism of glucosyl transfer appears feasible through the sp2 to sp3 hybridization changes in the carbon and nitrogen atoms of the arginine guanidinium function. Such a rehybridization can be described as “structural pyramidalization,” which enhances the nucleophilicity of nitrogen atoms.

The concept of enzymatic electrophilic functionalization of the arginine guanidinium group, omitting its deprotonation, was developed in studies on arginine kinases by Falcioni et al. [42]. These authors proposed a sophisticated mechanism of Arg phosphorylation, involving a process they described as “polarization–pyramidalization” of nitrogen in the arginine side chain. They also suggested that this phenomenon is exploited by many classes of enzymes mediating the post-translational modification of arginine, including N-glycosylation [42].

The presented series of transformations proceeding in a gas phase and resulting in arginine residue N-glucosylation appears analogous to the arginine deiminase mode of action. Arginine deiminase (EC 3.5.3.6) uses the catalytic Cys406 as an essential nucleophile to form an intermediate covalent adduct with the guanidinium carbon of the substrate, followed by ammonia elimination [43]. Other less adequate examples exist of liquid phase reactions employing protonated guanidine derivatives and thiolates to perform particular target-directed transformations [44]. It has also been reported that the arginine guanidinium ion reacts with a carbanion derived from the condensation of glutaraldehyde with the lysine ε-amine group [45].

2. Route B: Cysteine thiolate behaves as a catalytic base

In the alternative route B, the cysteine thiolate in the [Glc+/anh-Glc+][: S-Cys][Peptide]^z+^_OH_ ion pair deprotonates a conformationally accessible guanidinium moiety of protonated ArgH+ (Figure 6d). The resulting (unprotonated) guanidino group, while retaining its planar geometry, acquires nucleophilic properties. Next, it attacks the proximate Glc+ ion to form a charged N-glucosylated Arg+ (Figure 6c). In this variant of the arginine N-glycosylation mechanism, the basicity of the thiolate in a gas phase dictates its role as an initial proton acceptor triggering the subsequent transformation steps. 

Some experimental facts support this hypothetical sequence of rearrangements. In a liquid phase, the Arg of pKa 13.8 [46] can be N-functionalized using a stronger base—e.g., the Barton base of pKa 15.3 [47,48]. Analogously, with NCS reagent in a gas phase, N-acylation of the peptidyl arginine residue was observed exclusively if it existed in an un-protonated form [29,49]. It has also been reported that the pKa parameter, calculated for the cysteine residues present in some human kinases, ranges in scope from 7 to even 24 units [50,51]. This indicates enormous differences in the reactivity of the cysteine thiols dictated by the protein cavity characteristics. Thus, in favorable surroundings, cysteine can be much more basic than arginine. Studies on the *Rhodobacter sphaeroides* mitochondrial proton pump provide evidence of the role of Cys-139 as an initial proton acceptor acting in the altered form of Cytochrome c oxidase (CcO) [52]. The cited scientific data demonstrate the experimental conditions in which the [:S-Cys]Peptide thiolate behaves either as a strong nucleophile or as a super-basic proton acceptor.

### 3.3. Gas Phase Amadori Rearrangement

**Figure 7:**
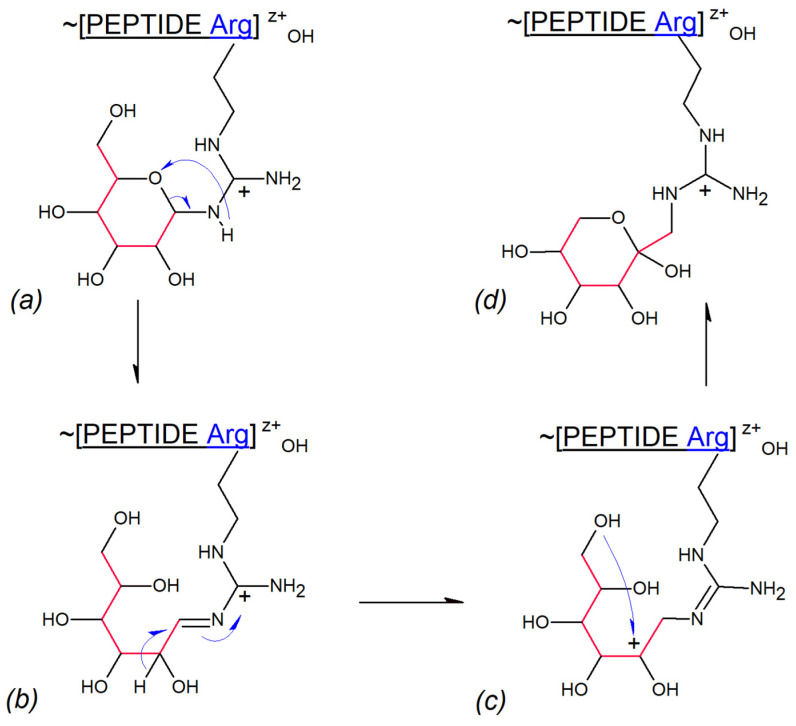
Hypothetical mechanism of Amadori rearrangement in gas phase. (**a**) Collisional pyranose ring opening within N-glucosylated peptidyl arginine (Arg+) leading to glucose-Schiff’s base formation; (**b**) C(2)→C(1) hydride ion shift leading to C(2)+ cationic intermediate; (**c**) N-fuctosyl ring closure involving C(6)-OH and C(2)+ cationic center; (**d**) N-fructosylated peptidyl arginine (Arg+) formation.

We demonstrated in the results section that Lys and Arg N-glucosylated gas phase transfer products experience Amadori rearrangement to N-fructosylated structures. To the best of our knowledge, such events have not been observed previously. The hypothetical mechanism of these transformations is depicted in Figure 7. The process is initiated by the collisional splitting of the C(5)O-C(1) bond in the glucose hemiaminal (Figure 7a), leading to the formation of the guanidinium Schiff base (Figure 7b). Hydride 1,2-migration from C(2) to C(1) results in a new cationic species (Figure 7c). Subsequent ring closure involving the C(6)-OH and C(2)+ cationic centers produces N-fructosylated guanidinium residue of Arg (Figure 7d). The postulated mechanism seems compatible with the low proton mobility environment of a gas phase, although 1,2-hydride shift mechanisms have also been proposed to explain enzymatic glucose to fructose interconversion [53].

The 1,2-hydride shift explains the fragmentation pattern of the S-adenosyl-L-methionine observed during CID analysis [54]. Analogously, the same phenomenon is proposed for elucidating differences in CID fragmentation of L-leucine and L-isoleucine [55].

## 4. Materials and Methods

The fragmentation spectra used in this study were derived from a library reported in a previous study. The list of CID and HCD spectral libraries containing S-glucosylated peptides is accessible in [14] (Table 1), and their sequences can be viewed in the Supplementary Materials at https://link.springer.com/article/10.1007/s00726-022-03208-7 (on 1 July 2024).

### 4.1. MS/MS Spectra Acquisition

All CID and HCD fragmentation spectra were generated using an LTQ Orbitrap Velos (Thermo Fisher Scientific, Bergen, Norway) spectrometer working in the regime of data-dependent acquisition. The normalized collision energy was set to 30%. Dynamic exclusion was disabled. Raw instrument data (.RAW) were processed using the Byonic search engine (Protein Metrics Inc., Cupertino, CA, USA, v.0–25) against the Uni-Protaccession_p00698.decoys.fasta. Protein FDR was set to 1% FDR. All searches used a 6-ppm precursor and 20-ppm fragment ion tolerance for HCD, with 0.500 Da tolerance for CID fragmentation. All searches considered tryptic peptides with a maximum of two missed cleavages.

### 4.2. Manual Curation of Diagnostic Fragment Ions

The fragmentation pathways shown in Figure 1 display ions and their lost or gained neutral fragments, the sizes of which were determined using a monoisotopic mass calculator accessible at https://www.sisweb.com/referenc/tools/exactmass.htm (accessed between 20 June 2022 to 31 January 2024). The results are presented in Table 8.

Theoretical values of y/b fragment ions for a particular peptide sequence were determined using an MS/MS fragmentation calculator provided by the University of Washington’s Proteomics Resource available online at https://proteomicsresource.washington.edu/cgi-bin/fragment.cgi (accessed between 20 June 2022 to 31 January 2024). The obtained data were used to calculate theoretical values for the target diagnostic fragment ions listed in Table 1, Section 2.2. Next, a manual search of selected MS/MS spectra was carried out to identify the *m*/*z* signals corresponding to particular diagnostic ions. The Excel sheets containing exemplary calculations and outcomes of manual spectra searches are also included in the Supplementary Materials (Supp Excel files).

## 5. Conclusions

The CID/HCD-fragmentation of S-glucopeptides is accompanied by a transfer of glucosyl ion Glc+ from cysteine to C-terminal Lys, Arg, and N-terminal amine groups. The moderate stability of the S-glycosidic bond under CID conditions enables the S-glucosylated precursor ions and their nascent fragment ions to play the double role of donor/acceptor in inter- and intramolecular glycosyl transfer processes.

We postulate that collisional activation of the S-glucosidic linkage may generate species carrying a reactive ion pair structure. The peptidyl thiolate component of the ion pair can display capabilities of both a super-active base and a strong nucleophile.

Such a duality can explain two alternative pathways of glycosyl transfer onto protonated arginine residue. While behaving as a nucleophile, the peptidyl thiolate attacks the guanidinium carbon, neutralizes its positive charge, and drives changes in orbital hybridization—a process we termed “pyramidalization.” The resulting transient electron-rich tetrahedral structure captures a nearby Glc+ cation and is stabilized by peptidyl thiol elimination. Ultimately, a charged planar N-glucosylated arginine residue is formed.

Glycosylation of cysteine and arginine in proteins is a new research area. Therefore, the results of our work might become informative, inspiring, or useful for a wider group of glycoproteomic communities.

## Figures and Tables

**Table 1:** The set of diagnostic ions for investigating glycosyl transfer effects.

Mark	Ion Type (Notation)	Moiety Lost or Gained
162 NL	[M + zH^+^ − 162]/z	1,2-anhydroglucose loss
NL ions	[M + zH^+^ − 180; −150; −120; −90]/z	hexose, pentose, tetrose, triose loss, respectively
179 NL	[M + zH^+^ − 179]/z	aminoglucose/aminofructose loss
204 NL	[M + zH^+^ − 204/z	1,2-anhydroglucose loss + carbodiimide loss
NL ions	[M + zH^+^ − nH_2_O]/z, *n* = 1, 2, 3	water loss, −18 Da, −36 Da, −54 Da
162 NG	y+/b+ [+162]	glucosyl gain
144 NGetc.	y+/b+ [+144; +126; +108]	loss of 1, 2, and 3 water molecules from 162 NG ions
78 NGetc.	y+/b+ [+78; +72; +42]	loss of (3H_2_O + CH_2_O), triose, tetrose from 162 NG ions

**Table 2:** The set of fragment ions from the +2 charged precursor, as evidence of fragmentations following paths A, B, and C.

[b-3H_2_O]+		b1	b2	b3	b4	b5	b6	b7	b8	b9	b10		0.20	Path C
[b-2H_2_O]+			b2	b3	b4	b5	b6	b7	b8	b9	b10		0.82	
[b-H_2_O]+			b2	b3							b10		0.03	
[b-150]+				b3	b4	b5	b6		b8	b9			0.004	Path B
[b-90]+			b2	b3	b4	b5	b6			b9	b10		0.22	
[b-120]+			b2	b3		b5	b6	b7		b9	b10		0.24	Path A
**[b]+**		**b1**	**b2**	**b3**	**b4**	**b5**	**b6**	**b7**	**b8**	**b9**	**b10**	**#**	1.00 Total abundance	
Total abundance 1.00		**C^Glc^**	**K**	**G**	**T**	**D**	**V**	**Q**	**A**	**W**	**I**	**R**		
		#		**y9**	**y8**	**y7**	**y6**	**y5**	**y4**	**y3**	**y2**		**[y]+**	
Path A	0.02			y9		y7		y5		y3			[y+162]+	
	0.10				y8	y7			y4	y3		y1	[y+42]+	
	0.04					y7	y6		y4	y3	y2		[y+24]+	
Path B	0.03			y9			y6		y4	y3		y1	[y+72]+	
	0.02				y8	y7		y5		y3	y2	y1	[y+12]+	
	0.01			y9						y3	y2	y1	[y+54]+	
Path C	0.03					y7			y4	y3			[y+144]+	
	0.04							y5	y4	y3	y2	y1	[y+126]+	
	0.03		y10	y9		y7			y4	y3	y2		[y+108]+	
	0.01								y4			y1	[y+78]+	

**Table 3:** The set of doubly glycosylated fragment ions and nascent diagnostic ions from the +2 charged precursor, as evidence of fragmentations following paths A, B, and C.

[b+108]+	b1		b3	b4	b5				b9			4.37	Path C
[b+126]+	b1		b3			b6	b7	b8	b9			0.28	
[b+144]+	b1		b3		b5							0.48	
[b+12]+			b3	b4	b5	b6		b8	b9			0.13	Path B
[b+72]+		b2	b3	b4	b5	b6			b9	b10		0.33	
[b+42]+		b2			b5	b6	b7		b9	b10		0.68	Path A
**[b+162]+**		**b2**	**b3**					**b8**				**1.00** Total abundance	
	**C^Glc^**	**K**	**G**	**T**	**D**	**V**	**Q**	**A**	**W**	**I**	**R**		

**Table 4:** Gas phase transformations of C[+162.]KGTDVQAWIR precursor of charge state +2.

Transformations	Diagnostic Ion(s)	Path
N-glucosylation of Arg	[y+162]+	A
N-glucosylation of Arg	[y+42; y+24]+	A
N-fructosylation of Arg	[y+72; y+54; y+12]+	B
N-fructosylation of Arg	[y+144; y+126; y+108; y+78]+	C
S→N (Glc+) migration and tetrose loss	[b-120]+	A
S→N (Glc+) migration and triose loss	[b-90]+	B
S→N (Glc+) migration and pentose loss	[b-150]+	B

**Table 5:** The set of fragment ions from the +3 charged precursor, as evidence of fragmentations following paths A, B, and C.

[b-3H_2_O]++			b2	b3	b4	b5	b6	b7			b10		0.15	Path C
[b-2H_2_O]++					b4		b6	b7			b10		0.10	
[b-H_2_O]++		b1		b3	b4	b5	b6	b7		b9			0.26	
[b-150]++							b6	b7		b9	b10		0.02	Path B
[b-90]++			b2	b3		b5	b6	b7		b9	b10		0.29	
[b-120]++					b4	b5	b6	b7			b10		0.14	Path A
**[b]++**						**b5**	**b6**	**b7**					**1.00**	
[b-3H_2_O]+		b1	b2	b3	b4	b5	b6		b8				0.16	Path C
[b-2H_2_O]+		b1	b2		b4	b5	b6						0.70	
[b-H_2_O]+		b1	b2			b5	b6	b7	b8				0.06	
[b-150]+			b2	b3	b4	b5	b6	b7					0.81	Path B
[b-90]+		b1	b2	b3		b5	b6	b7	b8				0.13	
[b-120]+		b1	b2	b3	b4	b5	b6	b7	b8				0.41	Path A
**[b]+**		**b1**	**b2**	**b3**	**b4**	**b5**	**b6**	**b7**	**b8**			**#**	**1.00** Total abundance	
Total abundance **1.00**		**C^Glc^**	**K**	**G**	**T**	**D**	**V**	**Q**	**A**	**W**	**I**	**R**		
		**#**				**y7**	**y6**	**y5**	**y4**		**y2**	**y1**	**[y]+**	
Path A	0.02									y3	y2		[y+162]+	
	0.06				y8			y5	y4	y3	y2	y1	[y+42]+	
	0.03				y8		y6	y5		y3	y2	y1	[y+24]+	
Path B	0.14								y4	y3			[y+72]+	
	0.07					y7	y6			y3	y2	y1	[y+12]+	
	0.02							y5				y1	[y+54]+	
Path C	0.02									y3	y2		[y+144]+	
	0.04									y3	y2	y1	[y+126]+	
	0.03									y3	y2	y1	[y+108]+	
	0.01									y3		y1	[y+78]+	
	**1.00**		**y10**			**y7**	**y6**	**y5**	**y4**	**y3**	**y2**		**[y]++**	
Path A	0.20		y10	y9	y8	y7		y5	y4		y2	y1	[y+162]++	
	0.17						y6	y5	y4	y3	y2		[y+42]++	
	0.12			y9	y8		y6	y5	y4	y3			[y+24]++	
Path B	0.10			y9			y6	y5	y4	y3		y1	[y+72]++	
	0.19		y10	y9	y8	y7	y6	y5					[y+12]++	
	0.08			y9			y6		y4				[y+54]++	
Path C	0.11				y8	y7			y4	y3	y2	y1	[y+144]++	
	0.17		y10	y9	y8	y7		y5		y3			[y+126]++	
	0.04		y10	y9		y7			y4		y2	y1	[y+108]++	
	0.14			y9	y8		y6	y5	y4		y2	y1	[y+78]++	

**Table 6:** The set of doubly glycosylated and nascent fragment ions from the +3 charged precursor, as evidence of fragmentations following paths A, B, and C.

[b+108]++	b1			b4	b5	b6	b7	b8	b9	b10		13.23	Path C
[b+126]++		b2	b3	b4	b5	b6	b7	b8		b10		7.82	
[b+144]++		b2	b3	b4	b5		b7	b8	b9	b10		2.41	
[b+12]++		b2	b3	b4	b5			b8	b9			2.45	Path B
[b+72]++	b1	b2			b5	b6		b8	b9	b10		2.67	
[b+42]++				b4					b9			0.33	Path A
**[b+162]++**	**b1**	**b2**	**b3**			**b6**		**b8**	**b9**			**1.00**	
[b+108]+	b1	b2	b3	b4	b5							1.27	Path C
[b+126]+	b1		b3									1.64	
[b+144]+	b1	b2	b3		b5							0.57	
[b+12]+	b1	b2	b3	b4		b6						1.15	Path B
[b+72]+	b1			b4			b7					0.70	
[b+42]+	b1	b2				b6						0.89	Path A
**[b+162]+**	**b1**	**b2**	**b3**	**b4**	**b5**							**1.00** Total abundance	
	**C^Glc^**	**K**	**G**	**T**	**D**	**V**	**Q**	**A**	**W**	**I**	**R**		

**Table 7:** Gas phase transformations of the C[+162.]KGTDVQAWIR precursor of charge state +3.

Transformations	Diagnostic ion(s)	Path
N-glucosylation of Arg	[y+162]+/++	A
N-glucosylation of Arg	[y+42; y+24]+/++	A
N-fructosylation of Arg	[y+72; y+54; y+12]+/++	B
N-fructosylation of Arg	y+144; y+126; y+108; y+78	C
S→N (Glc+) migration and tetrose loss	[b-120]+/++	A
S→N (Glc+) migration and triose loss	[b-90]+/++	B
S→N (Glc+) migration and pentose loss	[b-150]+/++	B

**Table 8:** List of mass losses and gains for precursors and fragment ions.

Entry	Notation	Monoisotopic Mass [Da]	Chemical Formula	Chemical Formula Calculation
1	204	204.074623	C_7_H_12_O_5_N_2_	1,2-anhydro-Glc + carbodiimide
2	180	180.063390	C_6_H_12_O_6_	Glc
3	179	179.079374	C_6_H_13_O_5_N	1-amino-Glc
4	162	162.052824	C_6_H_10_O_5_	1,2-anhydro-Glc
5	150	150.052824	C_5_H_10_O_5_	pentose
6	144	144.042260	C_6_H_8_O_4_	162 − H_2_O
7	126	126.031695	C_6_H_6_O_3_	162 − 2H_2_O
8	120	120.042260	C_4_H_8_O_4_	tetrose
9	108	108.021130	C_6_H_4_O_2_	162 − 3H_2_O
10	90	90.031695	C_3_H_6_O_3_	triose
11	78	78.010565	C_5_H_2_O	162 − 3H_2_O − CH_2_O
12	72	72.021130	C_3_H_4_O_2_	162 − triose
13	54	54.031695	H_6_O_3_	3H_2_O
14	54	54.010565	C_3_H_2_O	162 − triose − H_2_O
15	42	42.021798	CH_2_N_2_	carbodiimide
16	42	42.010565	C_2_H_2_O	162 − tetrose
17	36	36.021130	H_4_O_2_	2H_2_O
18	24	24.000000	C_2_	162 − tetrose − H_2_O
19	18	18.010565	H_2_O	H_2_O
20	12	12.00000	C	C

## Data Availability

Data is contained within the article and Supplementary Materials.

## Supplementary Materials

The following supporting information can be downloaded at: https://www.mdpi.com/article/10.3390/ijms25137483/s1.

## List of Abbreviations

Abbreviation	Meaning
anh-Glc	1,2-anhydroglucose
anh-Glc+	protonated 1,2-anhydroglucose
Arg-	arginyl residue
Arg+	charged peptidyl arginine
ArgH+	protonated peptidyl arginine
Asn-	asparaginyl residue
-C[S-Glc]	S-glucosylated peptidyl cysteine residue
CID	collision induced dissociation
Cys-	peptidyl cysteine
ESI	electrospray ionization
Fru-	fructosyl residue
Glc-	glucosyl residue
Glc+	glucose oxocarbenium ion
GlcNAc-	N-Acetylglucosamine residue
HCD	higher energy collisional dissociation
LC	liquid chromatography
Mono-ADP-ribosyl	mono(adenosine diphosphate [ADP]–ribosyl residue
MS	mass spectrometry
MS/MS	tandem mass spectrometry
NG	neutral gain
NL	neutral loss
Rha-	rhamnosyl residue
