# Supplementary material for: Evidence of Gas Phase Glucosyl Transfer and Glycation in the CID/HCD-Spectra of S-Glucosylated Peptides
Source: Int J Mol Sci. 2024 Jul 8;25(13):7483. doi: 10.3390/ijms25137483 (PMC11242366; doi:10.3390/ijms25137483)
Supplement: Supplementary file 1 [file ijms-25-07483-s001.zip › Buchowiecka Alicja_ SUPLEMENTARY MATERIAL.pdf]

Evidence of Gas Phase Glucosyl Transfer and Glycation in the CID/HCD-Spectra of S-Glucosylated Peptides

Alicja K. Buchowiecka

Institute of Molecular and Industrial Biotechnology, Faculty of Biotechnology and Food Sciences, Lodz University of Technology, Stefanowskiego 2/22, 90-537 Łódź, Poland; alicja.buchowiecka@p.lodz.pl

Supplementary material

Content

1. Figure S1: CID MS/MS spectrum of doubly charged C[+162]KGTDVQAWIR _ (PID 1603)
2. Figure S2: CID MS/MS spectrum of doubly charged C[+162]KGTDVQAWIR_ (PID 1603); 610-680 m/z range
3. Figure S3: CID MS/MS spectrum of triply charged C[+162]KGTDVQAWIR_ (PID 1606)
4. Figure S4: CID MS/MS spectrum of triply charged C[+162]KGTDVQAWIR_ (PID 1606); 405-455 m/z range
5. Figure S5: CID MS/MS spectrum of doubly charged C[+162]ELAAAMK_ (PID 222).
6. Figure S6: CID MS/MS spectrum of doubly charged C[+162]ELAAAMK_ (PID 222); 371-471 m/z range.
7. Figure S7: CID MS/MS spectrum of doubly charged RC[+162]ELAAAMK_ (PID 745).
8. Figure S8: CID MS/MS spectrum of doubly charged RC[+162]ELAAAMK_ (PID 745); 465-565 m/z range.
9. Figure S9: CID MS/MS spectrum of doubly charged WWC[+162.]NDGR_ (PID 1189).
10. Figure S10: CID MS/MS spectrum of doubly charged WWC[+162.]NDGR_ (PID 1189); 425-525 m/z range.
11. Figure S11: HCD MS/MS spectrum of doubly charged C[+162.]KGTDVQAWIR_ (PID 18335).
12. Figure S12: HCD MS/MS spectrum of triply charged C[+162.]KGTDVQAWIR_ (PID 18338).
13. Table S1: The set of fragment ions from the +2 charged precursor C[+162]ELAAAMK_ (PID 222), as evidence of CID fragmentations following paths A, B, and C.
14. Table S2: The set of doubly glycosylated fragment ions and nascent diagnostic ions from the +2 charged precursor C[+162]ELAAAMK_ (PID 222), as evidence of CID fragmentations following paths A, B, and C.
15. Table S3: The set of fragment ions from the +2 charged precursor RC[+162]ELAAAMK_ (PID 754), as evidence of CID fragmentations following paths A, B, and C.

16. Table S4: The set of fragment ions from the +2 charged precursor WWC[+162.]NDGR_ (PID 1189), as evidence of CID fragmentations following paths A, B, and C.
17. Table S5: The set of fragment ions from the +2 charged precursor SLGNWVC[+162]AAK_ (PID 1684), as evidence of CID fragmentations following paths A, B, and C.
18. Table S6: The set of fragment ions from the +2 charged precursor C[+162.]KGTDVQAWIR_(PID 18335), as evidence of HCD fragmentations following paths A, B, and C.
19. Table S7: The set of doubly glycosylated fragment ions and nascent diagnostic ions from the +2 charged precursor C[+162.]KGTDVQAWIR _ (PID 18335), as evidence of HCD fragmentations following paths A, B, and C. No diagnostic signals were detected.
20. Table S8: The set of fragment ions from the +3 charged precursor C[+162.]KGTDVQAWIR _ (PID 18338), as evidence of HCD fragmentations following paths A, B, and C.

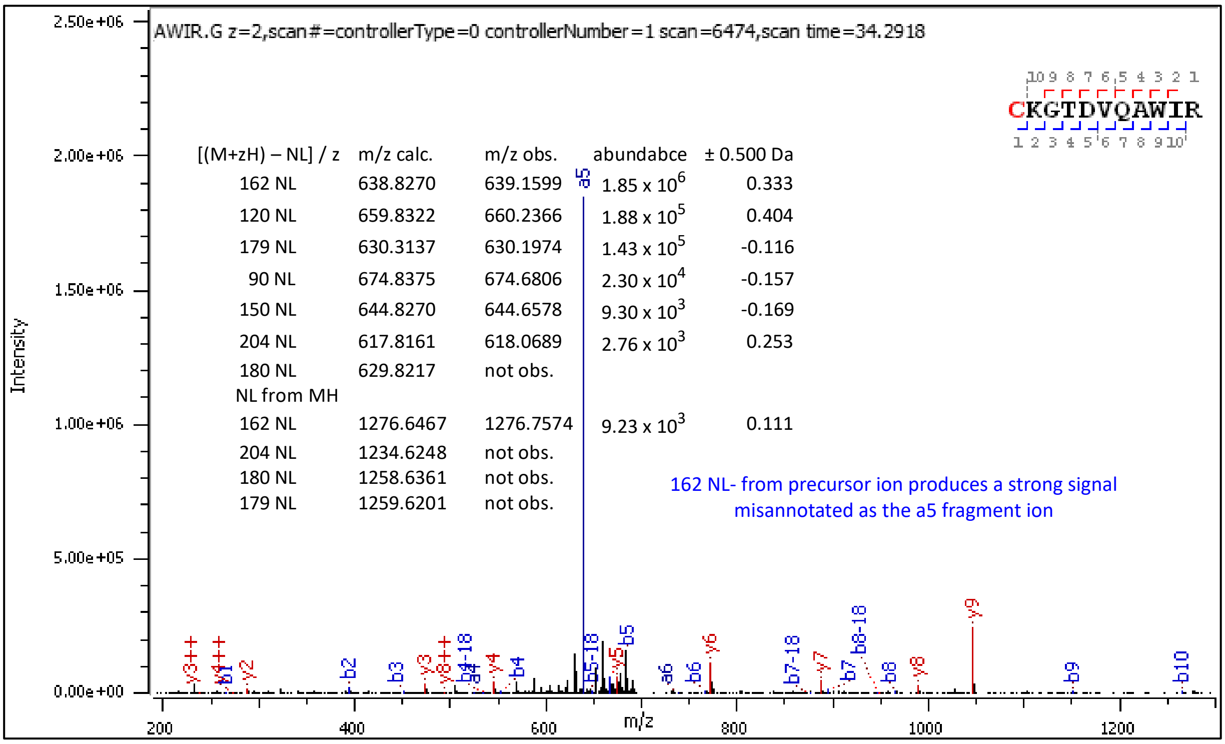

Figure S1. CID MS/MS spectrum of doubly charged S-glucosylated peptide **PID 1603_** C[+162]KGTDVQAWIR

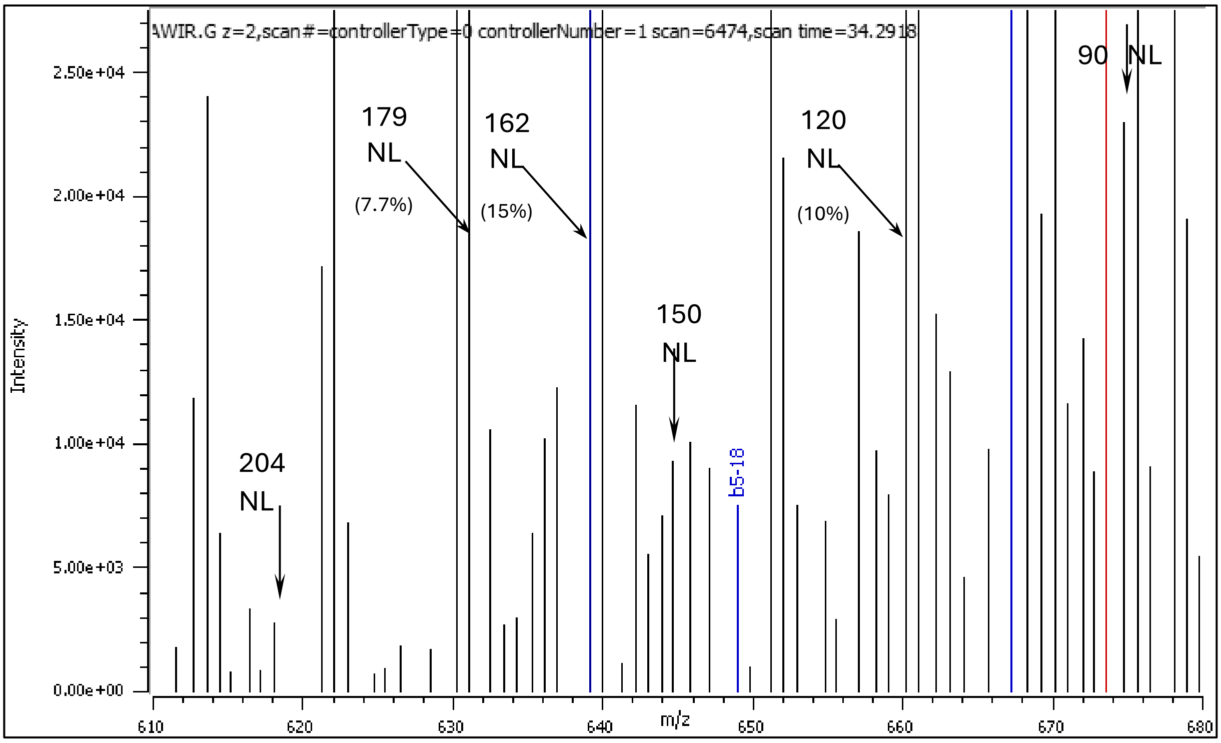

Figure S2. CID MS/MS spectrum of doubly charged S-glucosylated peptide **PID 1603_** C[+162]KGTDVQAWIR; 610-680 m/z range.

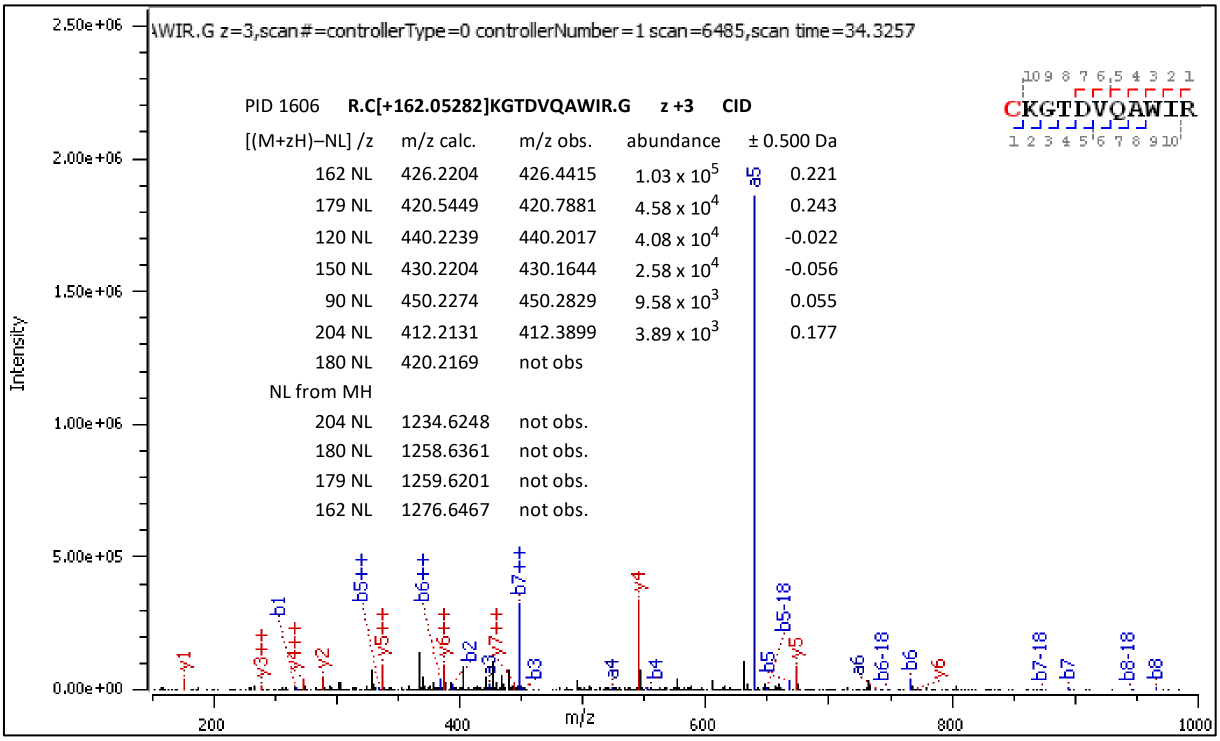

Figure S3. CID MS/MS spectrum of triply charged C[+162]KGTDVQAWIR_**PID 1606**

c

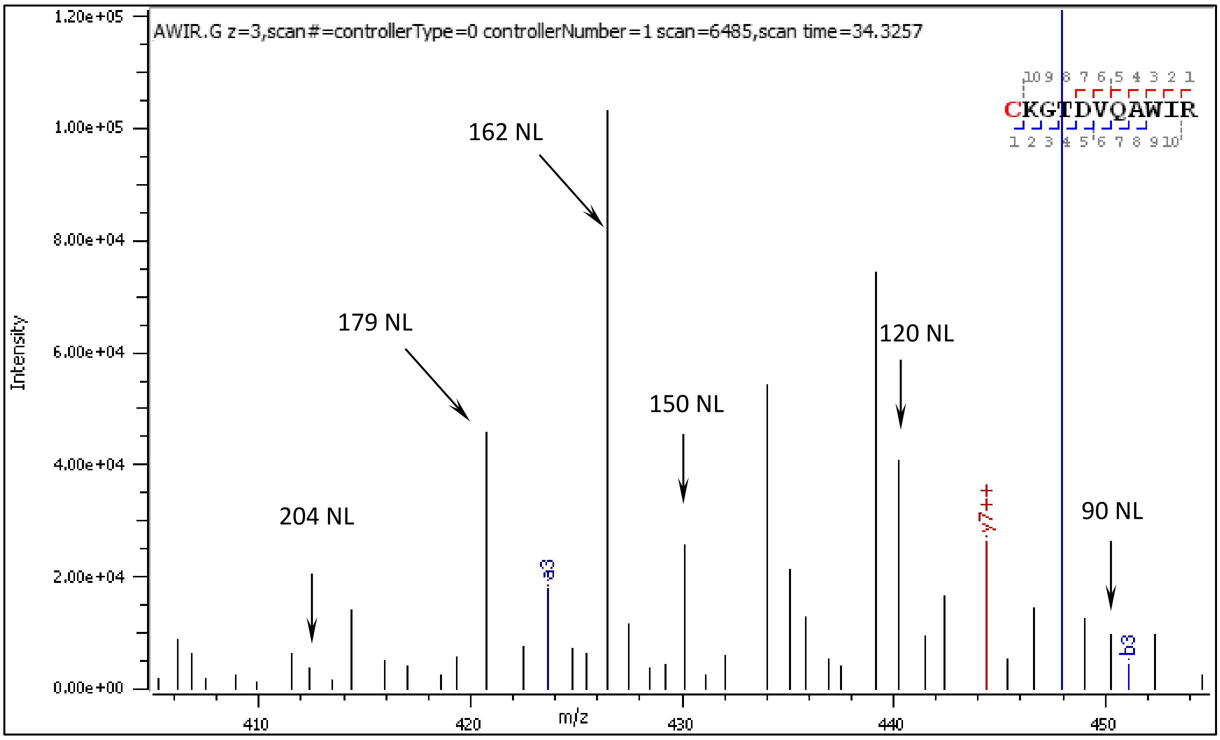

Figure S4. CID MS/MS spectrum of triply charged C[+162]KGTDVQAWIR_**PID 1606**;
405-455 m/z range

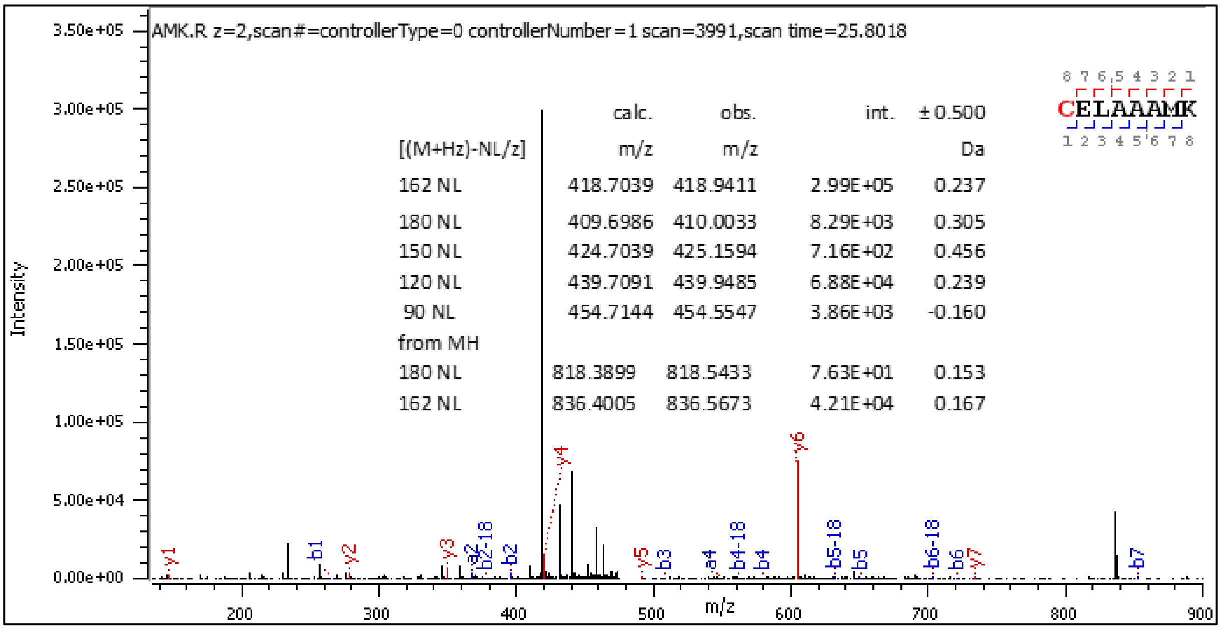

Figure S5. CID MS/MS spectrum of doubly charged S-glucosylated peptide **PID 222_** C[+162]ELAAAMK.

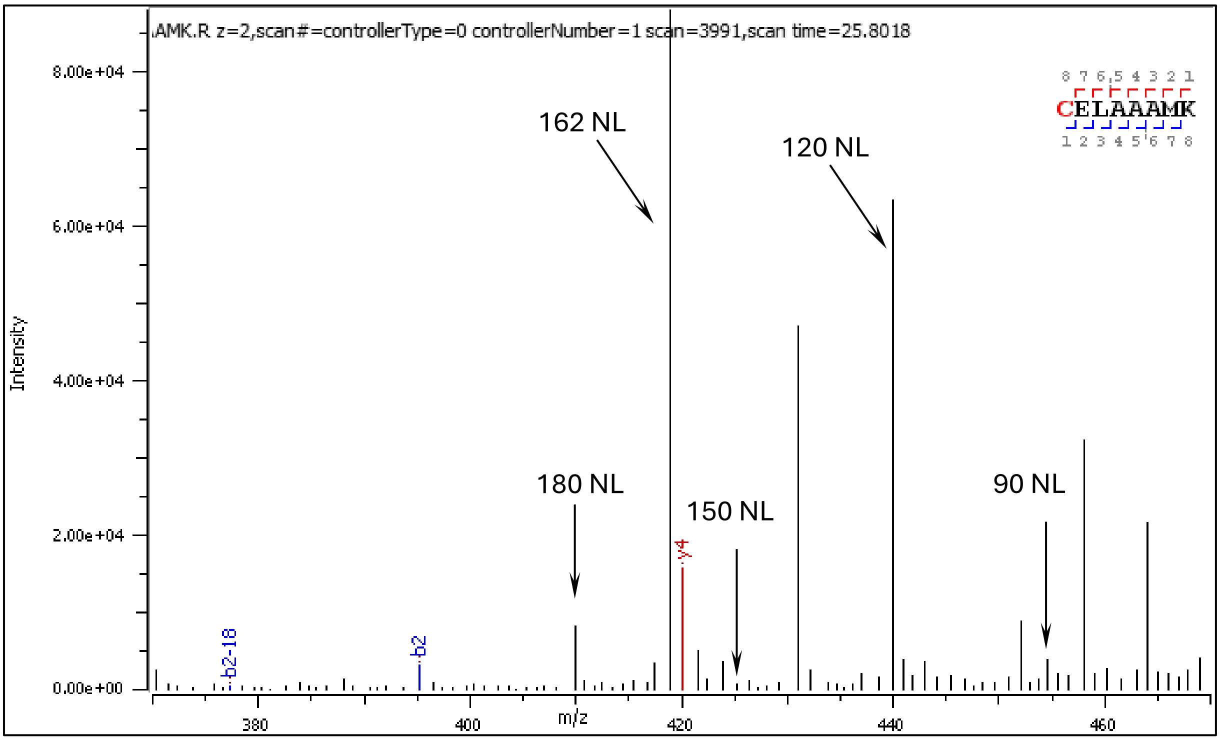

Figure S6. CID MS/MS spectrum of doubly charged S-glucosylated peptide **PID 222_** C[+162]ELAAAMK; 370 – 470 m/z range.

Table S1. The set of fragment ions from the +2 charged precursor C[+162]ELAAAMK_ (**PID 222**), as evidence of CID fragmentations following paths A, B, and C.

[b-84]+ *added*			b2	b3			b6	b7		0.41	Path C
[b-3H2O]+		b1	b2	b3	b4		b6	b7		1.19	Path C
[b-2H2O]+		b1	b2	b3	b4	b5	b6	b7		2.89	Path C
[b-H2O]+			b2		b4	b5	b6			0.37	Path C
[b-150]+			b2	b3	b4	b5		b7		**0.69**	Path B
[b-90]+			b2			b5	b6			0.26	Path B
[b-120]+			b2	b3	b4	b5	b6	b7		2.01	Path A
**[b]+**		**b1**	**b2**	**b3**	**b4**	**b5**	**b6**	**b7**		**1.00 Total abundance**	
**Total abundance**		**C**[Glc]	**E**	**L**	**A**	**A**	**A**	**M**	**K**		
	**1.00**	#	**y7**	**y6**	**y5**	**y4**	**y3**	**y2**	**y1**	**[y]+**	
Path A	0.66		y7		y5	y4		y2	y1	[y+162]+	
Path A	0.02			y6			y3	y2	y1	[y+42]+	
Path B	0.06			y6		y4	y3	y2		[y+72]+	
Path B	0.04			y6		y4	y3	y2		[y+12]+	
Path C	0.02		y7			y4	y3	y2		[y+144]+	
Path C	0.01		y7	y6		y4	y3	y2		[y+126]+	
Path C	0.01		y7	y6	y5	y4		y2	y1	[y+108]+	
Path C	0.01				y5		y3	y2		[y+78]+	

Table S2. The set of doubly glycosylated fragment ions and nascent diagnostic ions from the +2 charged precursor C[+162]ELAAAMK_ (**PID 222**), as evidence of CID fragmentations following paths A, B, and C.

[b+78]+		b2	b3	b4					2.63	Path C
[b+108]$^+$	b1		b3	b4					0.49	Path C
[b+126]$^+$	b1	b2	b3			b6			0.71	Path C
[b+144]$^+$	b1								4.36	Path C
[b+12]$^+$		b2	b3	b4	b5				**0.92**	Path B
[b+72]$^+$	b1	b2	b3	b4	b5				1.15	Path B
[b+42]$^+$		b2	b3	b4	b5	b6			2.55	Path A
**[b+162]$^+$**	**b1**	**b2**	**b3**						**1.00 Total abundance**	
	**C**[Glc]	**E**	**L**	**A**	**A**	**A**	**M**	**K**		

.

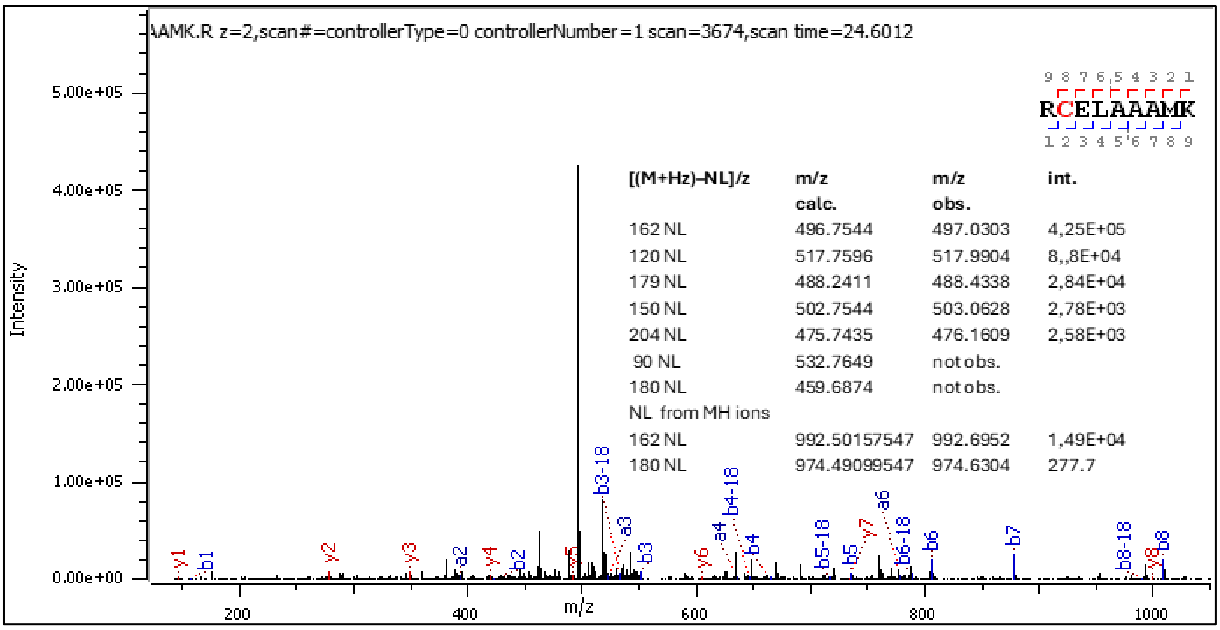

[(M+Hz)–NL]/z	m/z calc.	m/z obs.	int.
162 NL	496.7544	497.0303	4,25E+05
120 NL	517.7596	517.9904	8,,8E+04
179 NL	488.2411	488.4338	2,84E+04
150 NL	502.7544	503.0628	2,78E+03
204 NL	475.7435	476.1609	2,58E+03
90 NL	532.7649	not obs.	
180 NL	459.6874	not obs.	
NL from MH ions			
162 NL	992.50157547	992.6952	1,49E+04
180 NL	974.49099547	974.6304	277.7

Figure S7. CID MS/MS spectrum of doubly charged S-glucosylated peptide **PID 754**_RC[+162.]ELAAAMK.

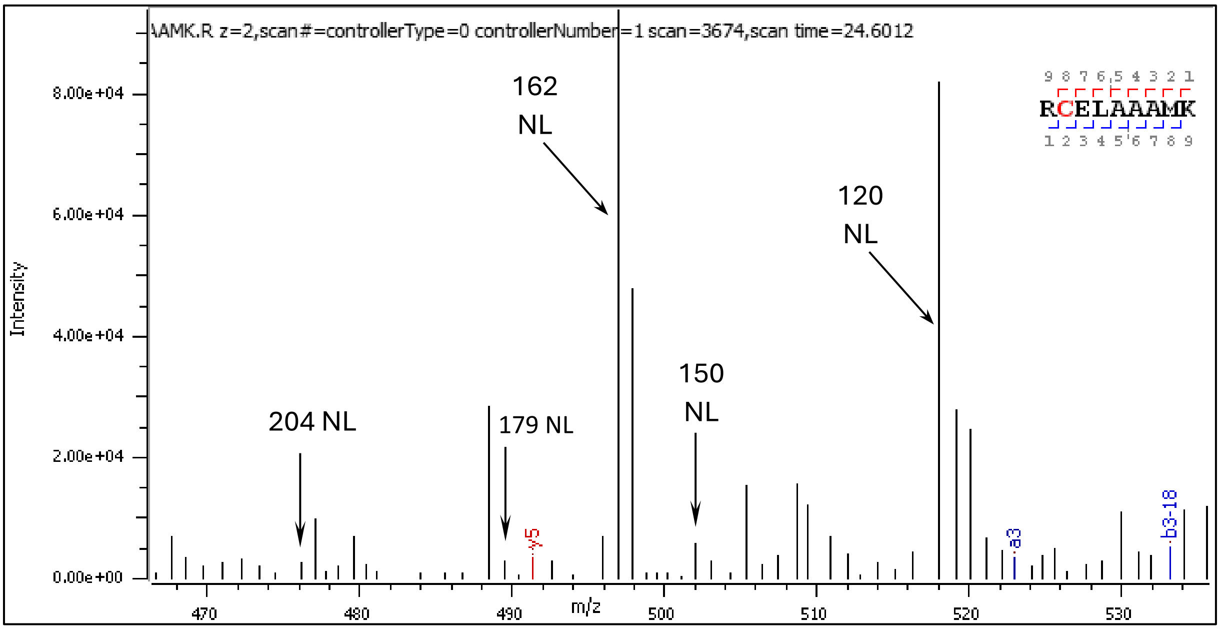

Figure S8. CID MS/MS spectrum of doubly charged S-glucosylated peptide RC[+162.]ELAAAMK (**PID 754)**; 465 – 565 m/z range

Table S3. The set of fragment ions from the +2 charged precursor RC[+162]ELAAAMK_ (**PID 754**), as evidence of CIDfragmentations following paths A, B, and C.

Fragment ions linked to double glycosylation are on green fields.

[b+78]+	b1	b2	b3	b4						2.63	Path C
[b+108]+	b1	b2		b4			b7			17.38	
[b+126]+	b1	b2	b3	b4	b5	b6		b8		10.36	
[b+144]+	b1		b3	b4	b5			b8		8.92	
[b+24]+		b2									
[b+54]+			b3								
[b+12]+	b1	b2		b4	b5		b7	b8		9.57	Path B
[b+72]+	b1	b2	b3	b4	b5	b6	b7			28.55	
[b+42]+		b2	b3	b4	b5	b6	b7			15.81	Path A
[b+162]				b4		b6	b7			total	1.00
[b-3H2O]+		b2			b5	b6		b8		0.03	Path C
[b-2H2O]+		b2	b3	b4		b6	b7			0.16	
[b-H2O]+		b2	b3	b4	b5	b6				0.17	
[b - 108]+ *intra-* **[b1+54]**											
[b - 138]+ *intra-* **[b1+24]**											
[b-150]+		b2		b4	b5	b6				0.04	Path B
[b-90]+		b2	b3		b5	b6	b7	b8		0.35	
[b-120]+			b3	b4	b5	b6	b7			0.01	Path A
**[b]+**	**b1**	**b2**	**b3**	**b4**	**b5**	**b6**	**b7**	**b8**		**1.00 Total abundance**	
**Total bundance 1.00**	**R**	**C^{Glc}**	**E**	**L**	**A**	**A**	**A**	**M**	**K**		
	#	**y8**	**y7**	**y6**	**y5**	**y4**	**y3**	**y2**	**y1**	**[y]+**	
**Path A** 0.38					y5	y4	y3	y2	y1	[y+162]+	
2.02		y8	y7			y4	y3		y1	[y+42] +	
**Paty B** 0.31			y7	y6		y4	y3	y2		[y+72]+	
0.44		y8	y7	y6	y5	y4	y3	y2	y1	[y+12]+	
**Path C** 0.34				y6	y5				y1	[y+144]+	
0.37				y6	y5	y4		y2	y1	[y+126]+	
0.27			y7	y6		y4		y2	y1	[y+108]+	
1.87						y4	y3		y1	[y+78]+	

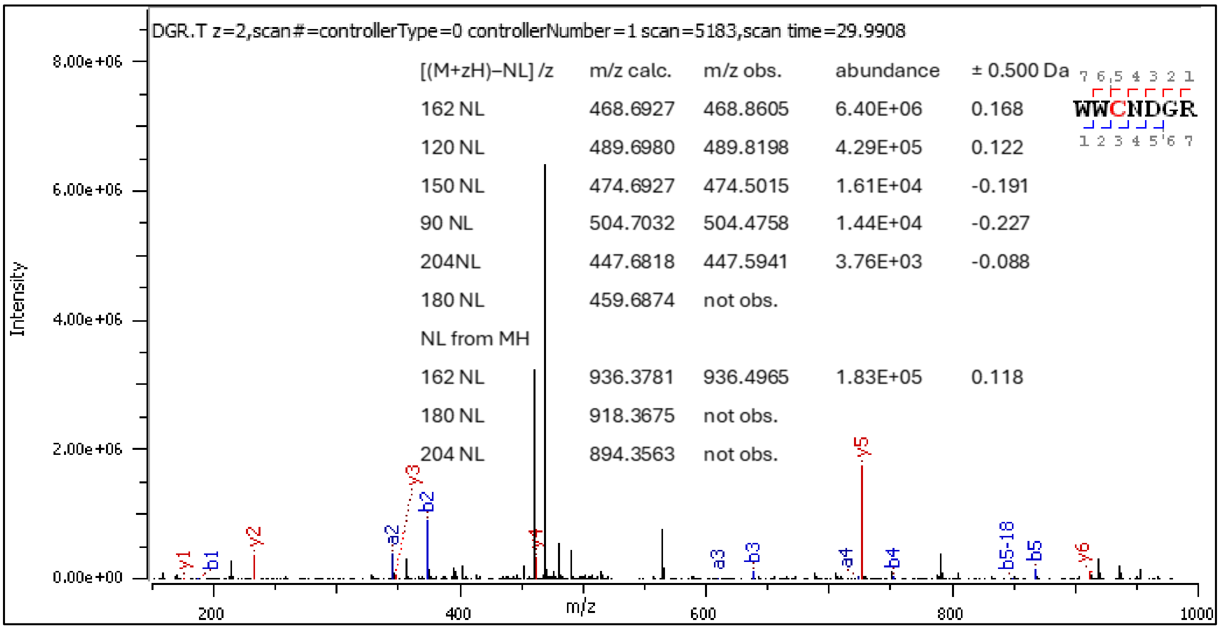

Figure S9. CID MS/MS spectrum of doubly charged S-glucosylated peptide **PID 1189_** WWC[+162.]NDGR

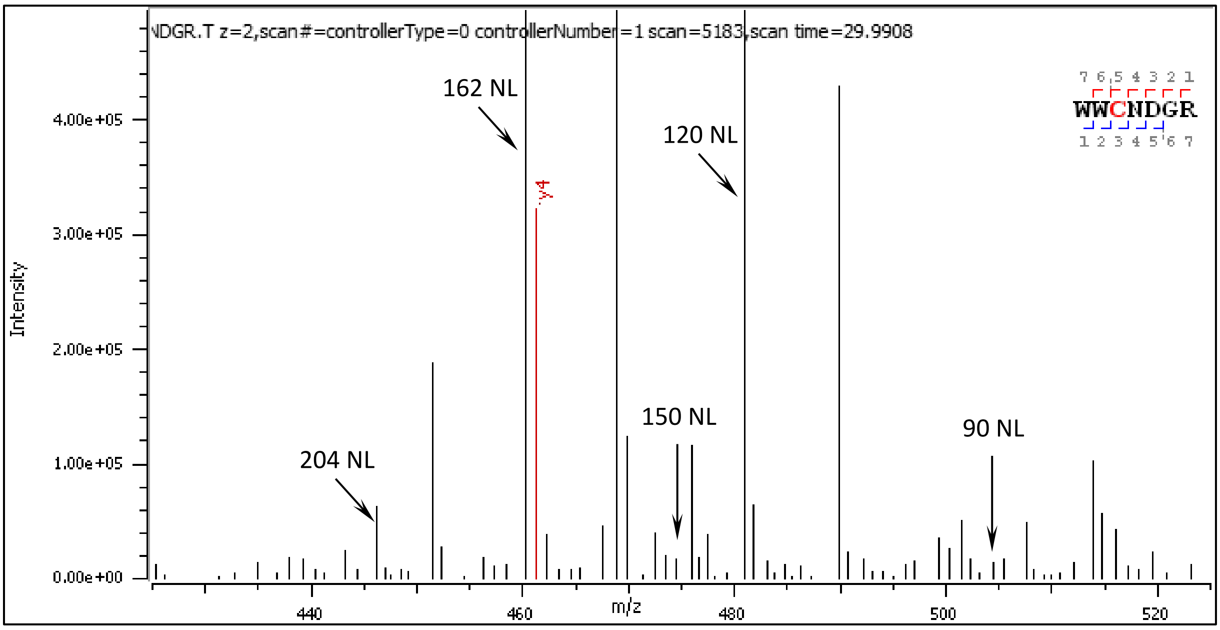

Figure S10. CID MS/MS spectrum of doubly charged S-glucosylated peptide **PID 1189_** WWC[+162.]NDGR; 425 -525 m/z range.

Table S4. The set of fragment ions from the +2 charged precursor WWC[+162.]NDGR _ (**PID 1189**), as evidence of CID fragmentations following paths A, B, and C.

Fragment ions linked to double glycosylation are on green fields.

$[b+108]^{+}$			b2							Path C
$[b+126]^{+}$		b1	b2							
$[b+144]^{+}$			b2							
$[b+12]^{+}$		b1				b5	b6			Path B
$[b+72]^{+}$		b1		b3						
$[b+42]^{+}$		b1		b3						Path A
**$[b+162]^{+}$**					**b4**				**1.00**	**Total ab.**
[b-3H2O]+					b4	b5	b6		0.003	Path C
[b-2H2O]+			b2	b3		b5	b6		0.02	
[b-H2O]+				b3	b4				0.01	
[b-150]+					b4	b5	b6		0.01	Path B
[b-90]+			b2	b3	b4	b5	b6		0.01	
[b-120]+				b3		b5			0.01	Path A
**[b]+**		**b1**	**b2**	**b3**	**b4**	**b5**		#	**1.00 Total abundance**	
**Total abundance 1.00**		**W**	**W**	**C^{Glc}**	**N**	**D**	**G**	**R**		
		#	**y6**	**y5**	**y4**	**y3**	**y2**	**y1**	**[y]+**	
Path A			y6	y5					[y-120]+	
Path B			y6						[y-90]+	
			y6						[y-150]+	
Path C			y6						[y-H2O]+	
									[y-2H2O]+	
			y6	y5					[y-3H2O]+	
	**1.00**			**y5**		**y3**	**y2**	**y1**	**[y+162]+**	
Path A	7794.00			y5	y4	y3	y2		[y+42]+	
	9076.30				**y4**		**y2**	**y1**	**[y+24]+**	
Path B	NA								[y+72]+	
	1.34			y5	y4	y3	y2		[y+12]+	
	2.32				**y4**			**y1**	**[y+54]+**	
Path C	1.87			y5	y4	y3	y2		[y+144]+	
	29849.70					y3	y2	y1	[y+126]+	
	6104.03			y5				y1	[y+108]+	
	0.55					y3	y2	y1	[y+78]+	

Table S5. The set of fragment ions from the +2 charged precursor SLGNWVC[+162]AAK_ (**PID 1684**), as evidence of CID fragmentations following paths A, B, and C.

Fragment ions linked to double glycosylation are on green fields.

[b+78]+			b2		b4	b5	b6	b7				1.32	Path C
[b+108]+			b2		b4	b5	b6	b7				1.56	
[b+126]+				b3	b4	b5	b6	b7				7.04	
[b+144]+		b1	b2	b3	b4	b5	b6	b7				15.24	
[b+12]+			b2	b3	b4	b5		b7		b9		4.65	Path B
[b+72]+		b1			b4	b5	b6	b7	b8			1.81	
[b+42]+			b2	b3	b4		b6			b9		1.02	Path A
[b+162]			b2	b3		b5	b6	b7				1.00	
[b-3H2O]+				b3		b5		b7	b8			0.01	Path C
[b-2H2O]+				b3		b5	b6	b7	b8	b9		0.08	
[b-H2O]+			b2	b3	b4	b5	b6	b7	b8	b9		0.26	
[b-150]+					b4	b5	b6	b7				0.10	Path B
[b-90]+				b3		b5	b6	b7	b8			0.11	
[b-120]+						b5	b6	b7	b8	b9		0.04	Path A
**[b]+**			**b2**	**b3**	**b4**	**b5**	**b6**	**b7**	**b8**	**b9**	#	**1.00** Total abundance	
Total abundance **1.00**		**S**	**L**	**G**	**N**	**W**	**V**	**C**GIc	**A**	**A**	**K**		
		#		**y8**	**y7**	**y6**	**y5**	**y4**	**y3**	**y2**		**[y]+**	
Path A	0.02		y9	y8	y7	y6	y5	y4				[y-120]+	
Paty B	0.04			y8		y6	y5	y4				[y-90]+	
	0.01		y9		y7	y6	y5					[y-150]+	
Path C	0.02			y8	y7	y6	y5	y4				[y-H2O]+	
	0.003						y5					[y-2H2O]+	
	0.01			y8	y7	y6		y4				[y-3H2O]+	
	1.00						y5	y4			y1	[y+162]+	
Path A	1.38				y7	y6	y5		y3		y1	[y+42] +	
Paty B	0.61			y8				y4	y3	y2	y1	[y+72]+	
	1.85				y7	y6	y5	y4	y3	y2	y1	[y+12]+	
Path C	0.62					y6	y5	y4	y3	y2		[y+144]+	
	0.62					y6	y5		y3	y2		[y+126]+	
	0.30					y6	y5		y3		y1	[y+108]+	
	0.51				y7			y4	y3	y2	y1	[y+78]+	

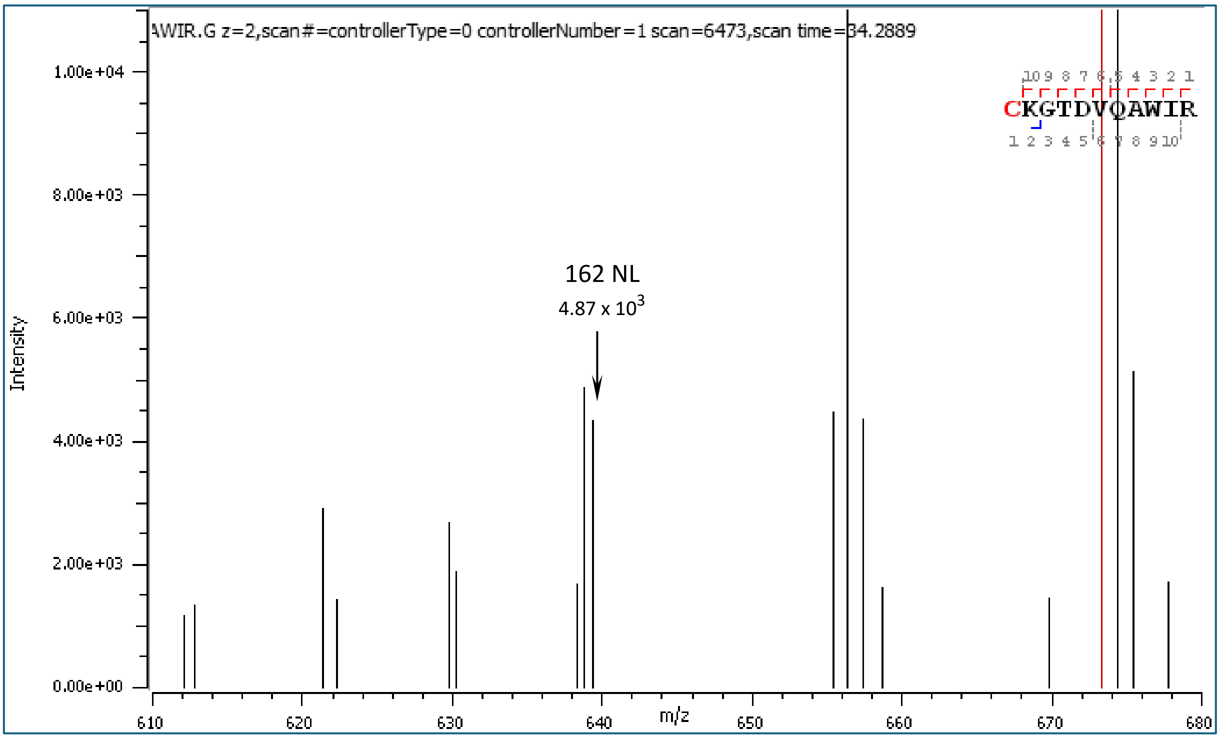

Figure S11. HCD MS/MS spectrum of doubly charged S-glucosylated peptide **PID 18335_** C[+162.]KGTDVQAWIR; 610-680 m/z range.

Table S6. The set of fragment ions from the +2 charged precursor C[+162.]KGTDVQAWIR _ (**PID 18335**), as evidence of HCD fragmentations following paths A, B, and C.

[b-3H2O]+			b2										2.08	Path C
[b-2H2O]+			b2	b3									3.45	
[b-H2O]+														
[b-150]+														Path B
[b-90]+														
[b-120]+														Path A
**[b]+**			**b2**									#	**1.00**	
**Total abundance***		C^Glc	K	G	T	D	V	Q	A	W	I	R	**Total abundance***	
	**1.00**	**#**	**y10**	**y9**	**y8**	**y7**	**y6**	**y5**	**y4**	**y3**	**y2**	**y1**	**[y]+**	
Path A													[y+162]+	
													[y+42]+	
													[y+24]+	
Path B													[y+72]+	
	0.002					y7							[y+12]+	
	0.010									y3			[y+54]+	
Path C													[y+144]+	
													[y+126]+	
													[y+108]+	
													[y+78]+	

Table S7. The set of doubly glycosylated fragment ions and nascent diagnostic ions from the +2 charged precursor C[+162.]KGTDVQAWIR _ (**PID 18335**), as evidence of HCD fragmentations following paths A, B, and C.

No diagnostic signals were detected.

$[b+108]^+$												
$[b+126]^+$												Path C
$[b+144]^+$												
$[b+12]^+$												Path B
$[b+72]^+$												
$[b+42]^+$												Path A
**$[b+162]^+$**												**0.00**
	**C^{Glc}**	**K**	**G**	**T**	**D**	**V**	**Q**	**A**	**W**	**I**	**R**	**Total abundance***

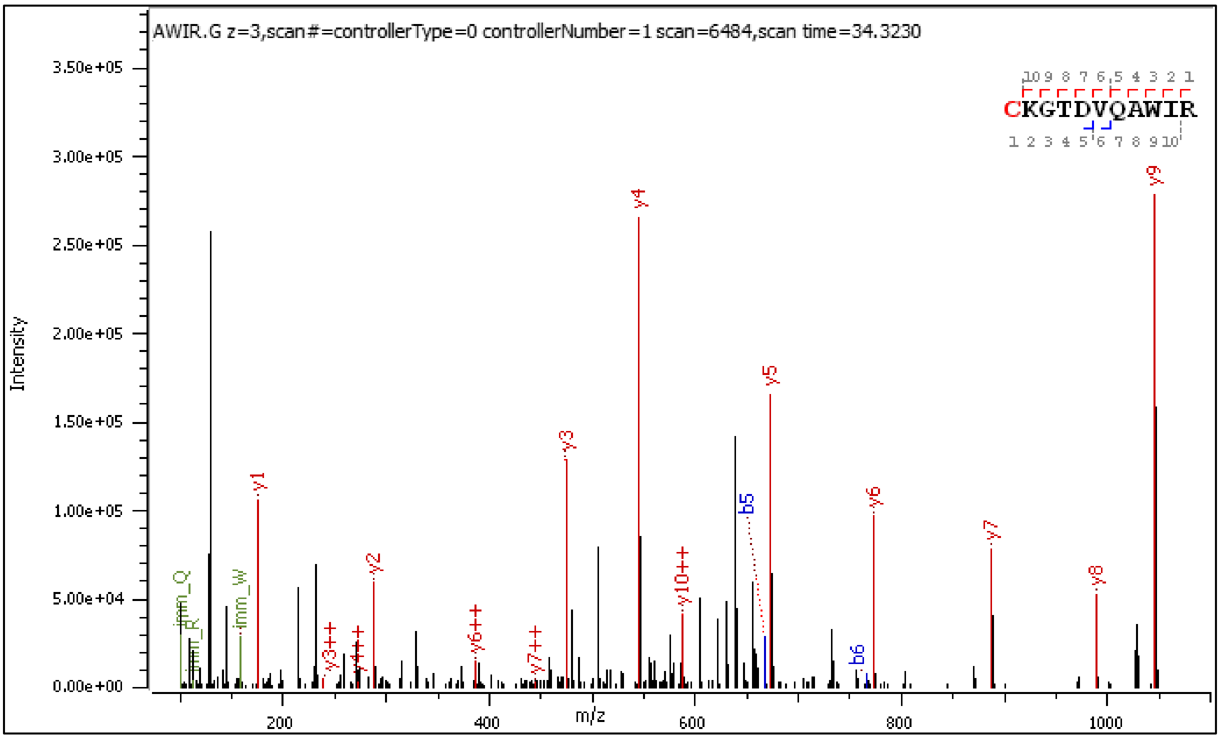

Figure S12. HCD MS/MS spectrum of triply charged S-glucosylated peptide **PID 18338_** C[+162.]KGTDVQAWIR; Neutral losses from molecular and precursor ions were not detected.

Table S8. The set of fragment ions from the +3 charged precursor C[+162.]KGTDVQAWIR _ (**PID 18338**), as evidence of HCD fragmentations following paths A, B, and C.

[b-3H2O]++														
[b-2H2O]++														Path C
[b-H2O]++										b9				
[b-150]++										b9				Path B
[b-90]++														
[b-120]++														Path A
**[b]++**														
[b-3H2O]+			b2			b5							0.19	
[b-2H2O]+			b2				b6						0.19	Path C
[b-H2O]+														
[b-150]+														Path B
[b-90]+														
[b-120]+						b5							0.12	Path A
**[b]+**			**b2**			**b5**	**b6**					#	**1.00**	
	**Total abundance**	**C[Glc]**	**K**	**G**	**T**	**D**	**V**	**Q**	**A**	**W**	**I**	**R**	**Total abundance**	
	**1.00**	#		**y9**	**y8**	**y7**	**y6**	**y5**	**y4**		**y2**	**y1**	**[y]+**	
													[y+162]+	
Path A	0.004				y8								[y+42]+	
													[y+24]+	
													[y+72]+	
Paty B													[y+12]+	
	0.02									y3		y1	[y+54]+	
													[y+144]+	
Path C													[y+126]+	
													[y+108]+	
													[y+78]+	
	**1.00**		**y10**			**y7**	**y6**		**y4**	**y3**			**[y]++**	
													[y+162]++	
Path A													[y+42] ++	
													[y+24]++	
													[y+72]++	
Paty B													[y+12]++	
	0.09								y4				[y+54]++	
													[y+144]++	
Path C													[y+126]++	
	0.12			y9									[y+108]++	
													[y+78]++	
